# Use of multivariate analysis to suggest a new molecular classification of colorectal cancer

**DOI:** 10.1002/path.4139

**Published:** 2013-01-25

**Authors:** Enric Domingo, Rajarajan Ramamoorthy, Dahmane Oukrif, Daniel Rosmarin, Michal Presz, Haitao Wang, Hannah Pulker, Helen Lockstone, Tarjei Hveem, Treena Cranston, Havard Danielsen, Marco Novelli, Brian Davidson, Zheng-Zhou Xu, Peter Molloy, Elaine Johnstone, Christopher Holmes, Rachel Midgley, David Kerr, Oliver Sieber, Ian Tomlinson

**Affiliations:** 1 Molecular and Population Genetics Laboratory Wellcome Trust Centre for Human Genetics University of OxfordUK; 2 Division of Surgery and Interventional Science University College London, Royal Free Hospital LondonUK; 3 Department of Pathology University College Hospital LondonUK; 4 Department of Oncology University of OxfordUK; 5 Oxford Medical Genetics Laboratories Churchill Hospital OxfordUK; 6 Bioinformatics Core Wellcome Trust Centre for Human Genetics OxfordUK; 7 Institute for Medical Informatics Oslo University HospitalNorway; 8 Centre for Cancer Biomedicine University of OsloNorway; 9 CSIRO Preventative Health Flagship North Ryde NSWAustralia; 10 Department of Statistics University of OxfordUK; 10 Nuffield Department of Clinical Laboratory Sciences University of OxfordUK; 12 Ludwig Colon Cancer Initiative Laboratory Ludwig Institute for Cancer Research MelbourneAustralia

**Keywords:** colorectal cancer, genetic pathways, clinico-pathological associations, prognostic markers, somatic mutations, molecular classification

## Abstract

**Abstract:**

Molecular classification of colorectal cancer (CRC) is currently based on microsatellite instability (MSI), KRAS or BRAF mutation and, occasionally, chromosomal instability (CIN). Whilst useful, these categories may not fully represent the underlying molecular subgroups. We screened 906 stage II/III CRCs from the VICTOR clinical trial for somatic mutations. Multivariate analyses (logistic regression, clustering, Bayesian networks) identified the primary molecular associations. Positive associations occurred between: CIN and TP53 mutation; MSI and BRAF mutation; and KRAS and PIK3CA mutations. Negative associations occurred between: MSI and CIN; MSI and NRAS mutation; and KRAS mutation, and each of NRAS, TP53 and BRAF mutations. Some complex relationships were elucidated: KRAS and TP53 mutations had both a direct negative association and a weaker, confounding, positive association via TP53–CIN–MSI–BRAF–KRAS. Our results suggested a new molecular classification of CRCs: (1) MSI^+^ and/or BRAF-mutant; (2) CIN^+^ and/or TP53^–^ mutant, with wild-type KRAS and PIK3CA; (3) KRAS- and/or PIK3CA-mutant, CIN^+^, TP53-wild-type; (4) KRAS^–^ and/or PIK3CA-mutant, CIN^–^, TP53-wild-type; (5) NRAS-mutant; (6) no mutations; (7) others. As expected, group 1 cancers were mostly proximal and poorly differentiated, usually occurring in women. Unexpectedly, two different types of CIN^+^ CRC were found: group 2 cancers were usually distal and occurred in men, whereas group 3 showed neither of these associations but were of higher stage. CIN^+^ cancers have conventionally been associated with all three of these variables, because they have been tested en masse. Our classification also showed potentially improved prognostic capabilities, with group 3, and possibly group 1, independently predicting disease-free survival. Copyright © 2012 Pathological Society of Great Britain and Ireland. Published by John Wiley & Sons, Ltd.

## Introduction

Two main molecular types of colorectal carcinoma (CRC) have been described, based on the 'molecular phenotypes' of chromosomal instability (CIN) and microsatellite instability (MSI or MIN). CIN is the more common and is generally detected by the presence of an abnormal chromosome complement or number (aneuploidy or polyploidy) 1997. MSI is the result of mismatch repair deficiency 1993, resulting in an increased mutation rate that is principally manifest as insertions and deletions in repetitive sequences. In terms of the somatic genetic pathways followed by MSI^+^ and CIN^+^ CRCs, there seems to be considerable functional overlap, but the specific mutations tend to differ: for example, MSI^+^ tumours tend to acquire mutations in *AXIN1*, *BRAF* and *BAX*, whereas CIN^+^ tumours have mutations in *APC*, *KRAS* and *TP53* 3. In addition, CIN^+^ and MSI^+^ tumours are associated with different clinico-pathological features: the former tend to be well/moderately differentiated and distal, and the latter poorly differentiated, proximal and more frequent in women 2. MSI is also an established marker of good prognosis. However, there remains considerable heterogeneity within the MSI^+^ or CIN^+^ groups. A third molecular phenotype, known as the CpG island methylator phenotype (CIMP), has also been described 3. CIMP is characterized by a high degree of age-independent methylation in gene promoters and tends to overlap with MSI, in part because promoter methylation of the mismatch repair gene *MLH1* is the most usual alteration leading to MSI 4.

The very concept of somatic genetic pathways implies that some mutations are co-selected, presumably as a result of variation in the cancer cell's microenvironment, one component of which is the pre-existing mutations in that cell. However, whilst some consistent pairwise associations between mutations in CRC have been identified, it is far from clear which mutations are co-selected and which are secondarily associated via other genetic changes. We hypothesized that some of the considerable residual heterogeneity in the behaviour of CRCs could be explained by refining the established genetic pathways of tumorigenesis and by identifying new ones. However, most previous studies, including our own, have used insufficient samples and/or analysed too few genes for this objective to be achieved 7–12. In this study, we analysed over 900 CRCs from the VICTOR clinical trial of stage II/III colorectal cancer. We profiled 11 somatic genetic alterations and performed multivariate analysis using regression, clustering and Bayesian network approaches. This strategy allowed us to better characterize the existing pathways of colorectal tumorigenesis, to find additional, less common pathways and to propose primary molecular determinants of tumour behaviour.

## Materials and methods

The VICTOR randomized trial of rofecoxib or placebo post-primary treatment recruited a total of 2434 stage II/III CRC patients between 2002 and 2004 11. Formalin-fixed, paraffin-embedded blocks were available for 965 of these patients. Haematoxylin and eosin (H&E)-stained sections were reviewed, and normal tissue and colorectal carcinoma within each section were identified. Samples from 59 patients were discarded because of lack of tumour, leaving 906 cancers (Table 1). All tumour samples were collected prior to non-surgical therapy, with the exception of 67 rectal carcinomas that had been treated with neo-adjuvant radiotherapy. Clinico-pathological variables at presentation were obtained from the trial database and treated as either binary [location (left versus right), sex, stage (II versus III)] or continuous [age, differentiation (well, moderate, poor)], as appropriate. Paired normal samples from 795 of these patients were obtained from additional blocks with only normal tissue (*n =* 479), blood sample (*n =* 244) or pure, clearly separate normal tissue in the same block as the tumour (*n =* 72). For DNA extraction, all the normal blocks and carcinomas with > 80% cancer cells were cut into scrolls. Other tumour blocks were cut into 10 µm sections and needle-microdissected, with an H&E section as a guide to ensure population purity. Tissues from scrolls and microdissections were digested with proteinase K and DNA was extracted using the DNeasy Kit (Qiagen). DNA from blood was extracted with the Maxwell 16 Blood DNA Purification Kit (Promega). For ploidy analysis, all carcinoma blocks were cut into a further scroll. All research was performed according to the tenets of the Declaration of Helsinki and approved by Oxfordshire Research Ethics Committee B (Approval No. 05\Q1605\66).

**Table 1:** Clinico-pathological profiles of the tumours used in the study

	**Number of tumours (%)**
Stage	
II	445 (49)
III	461 (51)
Gender	
Male	581 (64)
Female	325 (36)
Site	
Proximal/right	306 (35)
Distal/left	577 (65)
No info	23
Age (years)	
Mean (SD)	64.1 (10)
Median (range)	64.6 (24.6–86.3)
Differentiation	
Well	73 (8)
Moderate	725 (82)
Poor	88 (10)
No info	20

Mutation screening, microsatellite instability, LOH and CIN analysis used standard, previously described methods. These and the statistical methods are described in the Supplementary material.

## Results

### Baseline analysis of molecular associations

The set of 906 stage CRCs (Table 1) was analysed for CIN, MSI and almost all of the most common somatic mutations in colorectal cancer (*KRAS*, *NRAS*, *BRAF*, *PIK3CA*, *TP53* and *FBXW7*/*CDC4*). LOH analysis was performed in 795 tumours from which constitutional DNA was available, targeting chromosomes 5q near *APC*, 17p near *TP53* and 18q near *SMAD4*. A summary of the molecular findings is shown in Table 2. Overall, the frequencies of molecular alterations, the mutation spectra (see Supplementary material, Figure S1) and the pairwise associations (see Supplementary material, Tables S1, S2), were in good agreement with those previously established in the literature 4,6,12,14–19, and we shall not consider them further here. A new finding was that *FBXW7/CDC4* mutations were not associated with any other mutation or clinico-pathological variable. We also examined stage-specific associations, the only significant results being that stage III cancers tended to be CIN^+^.

**Table 2:** Overall frequencies of the molecular alterations analysed

Molecular alteration	Alteration frequency (%)
CIN	586/861 (68)
MSI	119/892 (13)
*KRAS*	304/898 (34)
*BRAF*	91/903 (10)
*NRAS*	32/869 (4)
*PIK3CA*	104/896 (12)
*TP53*	329/753 (44)
*FBXW7 (CDC4)*	35/750 (5)
5q LOH	160/566 (28)
18q LOH	334/694 (48)
17p LOH	344/666 (52)

180 (21% in total) CRCs were ‘double-negative’ (MSI^–^CIN^–^). In general, the double-negative tumours resembled MSI^–^CIN^+^ cancers more than MSI^+^CIN^–^ cancers, and we therefore compared MSI^–^CIN^–^ with MSI^–^CIN^+^ tumours. The MSI^–^CIN^–^ cancers presented at an earlier stage (102/180 versus 244/557 stage II, respectively; *p =* 0.003, *q =* 0.01) had lower frequencies of *TP53* mutation and 17p LOH, and showed a borderline association with *KRAS* mutation (see Supplementary material, Tables S2, S3). Logistic regression-based multivariate analysis showed that the MSI^–^CIN^–^ tumours remained associated only with lower stage and lack of *TP53* mutation (see Supplementary material, Table S3).

Twenty-three cancers (3%) were ‘double-positive’ (MSI^+^CIN^+^). Compared with all other cancers, the double-positive tumours tended to be right-sided (14/22 versus 278/804; *p =* 0.007, *q =* 0.03). No molecular alteration, including *TP53* mutation and 17p LOH, was significantly associated with this small group of tumours, although they had a relatively high frequency of *BRAF* mutation (see Supplementary material, Table S2). Overall, double-positive cancers appeared to resemble MSI^+^CIN^–^ cancers most closely.

### Searching for the primary intermolecular associations

In order to define new genetic pathways of colorectal carcinogenesis, we sought evidence for the primary drivers of the associations between somatic mutations (*KRAS*, *NRAS*, *BRAF*, *PIK3CA*, *TP53*, *FBXW7*), CIN, MSI and the three sites of LOH (see Supplementary material, Tables S2, S3). Approximately half of the associations found by pairwise analysis were no longer significant, suggesting that they were secondary to a primary association.

**Figure 1:**
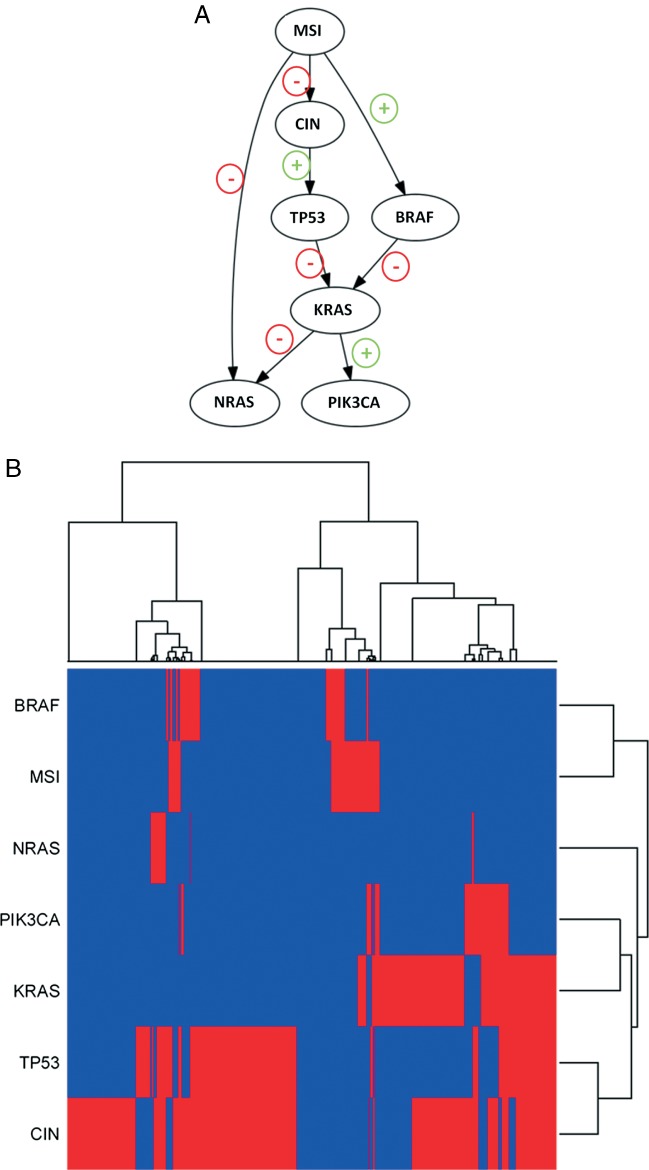
Multivariate analysis of seven molecular alterations in 705 tumours. (A) Bayesian network analysis. Edges represent conditional dependencies and nodes that are not connected represent variables which are conditionally independent of each other. Positive and negative associations have been marked as + and –, respectively. Note that the direction of the arrows is not an indication of causality. (B) Unsupervised hierarchical cluster analysis by tumour (horizontal) and mutation (vertical). Samples with and without alterations are marked in red and blue, respectively.

Since logistic regression analysis to identify the primary determinants of associations can be sensitive to small, chance associations or missing data when multiple highly correlated events occur, we additionally performed a Bayesian network analysis to detect primary associations among selected molecular variables (*KRAS*, *BRAF*, *NRAS*, *PIK3CA*, *TP53*, MSI, CIN) that had been successfully typed in the full dataset. *FBXW7* was excluded owing to its lack of any association, and the LOH events were omitted owing to their strong associations with each other, CIN and *TP53* mutation. The network analysis took the form of a probabilistic model that represents the conditional dependencies between random variables via a directed acyclic graph. We found that the primary positive associations were between: (a) CIN and *TP53*; (b) MSI and *BRAF*; and (c) *KRAS* and *PIK3CA*. The primary negative associations were between MSI and both CIN and *NRAS*, and between *KRAS* and each of *BRAF*, *NRAS* and *TP53*. These associations are shown in a simple, graphical form in Figure 1A. In almost all of these cases, the network analysis found the same primary associations as the logistic regression analysis (Figure 1A; see also Supplementary material, Table S3). Exceptions were the associations between *PIK3CA* mutation and MSI and between *PIK3CA* mutation and *TP53* that were only present in the logistic regression analysis (Figure 1A; see also Supplementary material, Table S3).

The network analysis additionally detected associations that were formally absent from the logistic regression, because those variables were dropped from the latter owing to excessive co-variation. A case in point was the primary negative associations between *NRAS* mutation and both *KRAS* mutation and MSI. The network analysis also showed the underlying reasons why some associations that were significant in the pairwise analysis were no longer significant in the multivariate logistic regression analysis. Examples included the negative associations between CIN and *BRAF* (which was secondary to associations with MSI) and between MSI and *TP53* (which was secondary to associations with CIN) (Figure 1A).

Of particular interest in the network analysis was the detection of association loops (Figure 1A). The loops between MSI and *NRAS* suggested two independent negative associations, one direct and the other indirect via CIN, *TP53* and *KRAS*. Most intriguing was the loop between MSI and *KRAS*. Here, there existed not only a direct negative association between *TP53* and *KRAS* mutations, but also a weaker, indirect positive association via CIN, MSI, *KRAS* and *BRAF*. The indirect positive association found by multivariate analysis was consistent with data in the literature from pairwise association testing, but the direct negative association was less well supported. We therefore performed a meta-analysis of other published studies 7,9,17,20–25 and this confirmed an overall pairwise negative *KRAS–TP53* mutation association (see Supplementary material, Table S4).

The logistic regression and network analyses had provided strong evidence to show which of the reported inter-molecular associations in CRC were primary and which were secondary (indirect). We undertook one further analysis to support our findings by performing unsupervised hierarchical clustering of the same alterations used in the network analysis. The cluster analysis fully supported the other two methods, indicating the four basic groups, as: *KRAS* and/or *PIK3CA*; CIN and/or *TP53*; MSI and/or *BRAF*; and *NRAS* (Fiugre 1B, vertical axis). Clustering of the tumours (Figure 1B, horizontal axis) showed them to be separated into two main groups, where the main discriminating molecular variable was *KRAS* mutation; all the mutants were present in the second cluster and none of the first cluster was mutant. Most *NRAS* and *PIK3CA* mutants were in the first and second clusters, respectively. CIN and *TP53* mutations were found in both clusters but were over-represented in the first one. MSI and *BRAF* mutations were present as subclusters within both of the two main clusters.

### Identifying groups of colorectal cancers based on shared molecular changes

We sought to identify groups of CRCs that would form the basis of our proposed molecular classification. The data suggested that this classification should be based not only on MSI, but also on *NRAS* mutation, on the negative association between *TP53* and *KRAS* mutations, and on CIN (Fiugre 1; see also Supplementary material, Table S3). Based on the primary positive and negative intermolecular associations described in the previous section, we chose CRC groups characterized by:

1. MSI and/or *BRAF* mutation.
2. CIN and/or *TP53* mutation, with wild-type *KRAS* and *PIK3CA*.
3. *KRAS* and/or *PIK3CA* mutation with CIN, but without *TP53* mutation
4. *KRAS* and/or *PIK3CA* mutation without CIN or *TP53* mutation.
5. *NRAS* mutation.

**Figure 2:**
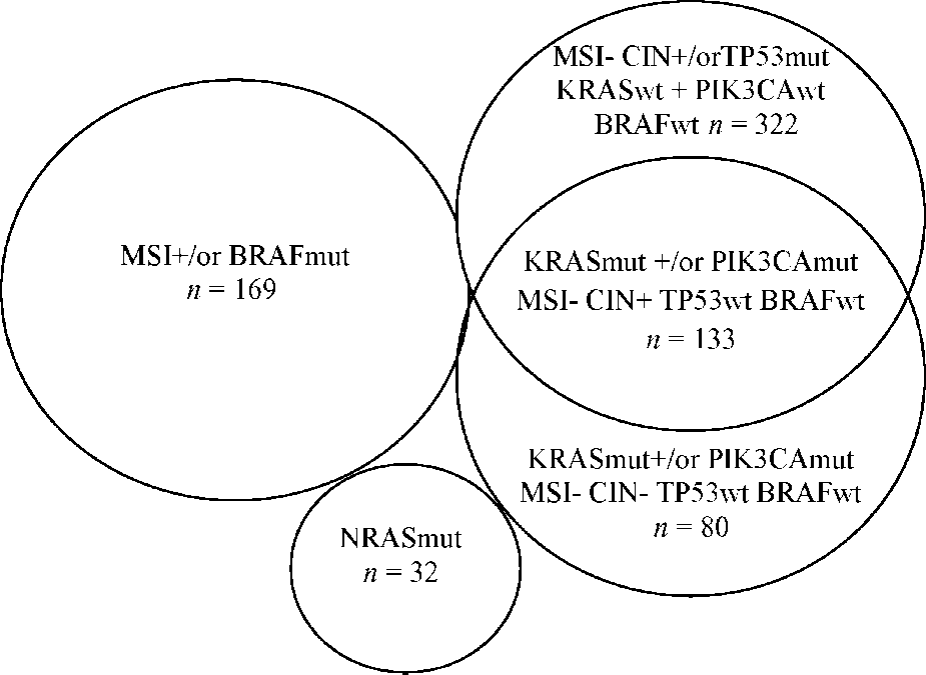
Proposed molecular classification of colorectal cancers. The proposed grouping is based on the following process. Initially, we utilized the near-complete lack of overlap between MSI^+^ and CIN^+^ cancers to identify two groups. Owing to the observed strong, primary associations, we then provisionally added BRAF-mutant tumours to the MSI^+^ group and TP53-mutant cancers to the CIN^+^ group. We retained the MSI^+^ and/or BRAF-mutant tumour group, irrespective of other genetic changes, since TP53, KRAS and NRAS mutations were uncommon in these cancers. We then formed a group of NRAS-mutant tumours, irrespective of their other genetic changes, since NRAS mutations were not positively associated with any other molecular variable. We next provisionally added a KRAS- and/or PIK3CA-mutant (but TP53-wild-type) group, owing to the negative association between KRAS and TP53. However, since we found no negative association between KRAS and CIN, we subdivided the KRAS- and/or PIK3CA-mutant group into CIN^+^ and CIN^–^ groups, leaving the great majority of TP53-mutant cancers in a CIN^+^, KRAS-wild-type and PIK3CA-wild-type group. In total, this classification encompasses 736/906 (81%) cancers. In addition, 78 double-negative cancers had no detected changes in KRAS, PIK3CA, NRAS, TP53 or BRAF. The remaining 92 cancers had a variety of 'atypical' mutation combinations.

These groups encompassed over 80% of all the CRCs studied. In addition, we proposed two further groups: group 6 with no detectable mutations; and a ‘miscellaneous’ group 7. Rationale for the grouping is provided in Figure 2.

### Association between CRC groups and clinico-pathological variables

Since it was hoped that the seven CRC groups would have clinical relevance, we used multiple logistic regression analysis to test the groups for associations with clinico-pathological variables (gender, age, tumour location, stage, grade, trial randomization arm, and treatment with chemotherapy or radiotherapy). Each group was tested in turn against all others, incorporating all variables in a reverse stepwise analysis (Table 3). Group 1 essentially included the MSI^+^ group of tumours and, as expected, these cancers were strongly associated with proximal location, poor differentiation and female gender.

**Table 3:** Associations between proposed molecular groups of colorectal cancers and clinico-pathological variables

Outcome variable	Description of group	Stage (II versus III)	Location (proximal versus distal)	Differentiation (grade, WD, MD, PD)	Gender (F versus M)
Group 1	MSI and/or BRAF-mutant	OR = 0.74, *p =* 0.12	**OR = 0.18, *p =* 3 × 10^–18^**	**OR = 2.08, *p =* 0.001**	**OR = 2.06, *p =* 2 × 10^-4^**
Group 2	CIN^+^ and/or TP53-mutant with WT KRAS and PIK3CA	OR = 0.95, *p =* 0.74	**OR = 3.09, *p =* 4 × 10^–11^**	OR = 1.03, *p =* 0.85	**OR = 0.62, *p =* 0.003**
Group 3	KRAS- and/or PIK3CA-mutant, CIN^+^, TP53-WT	**OR = 1.58, *p =* 0.02**	OR = 0.98, *p =* 0.91	OR = 0.74, *p =* 0.21	OR = 1.22, *p =* 0.3
Group 4	KRAS- and/or PIK3CA-mutant, CIN^–^, TP53-WT	OR = 0.85, *p =* 0.53	OR = 1.23, *p =* 0.44	OR = 0.86, *p =* 0.6	OR = 0.76, *p =* 0.29
Group 5	NRAS-mutant	OR = 1.03, *p =* 0.93	OR = 0.96, *p =* 0.93	OR = 0.89, *p =* 0.78	OR = 0.93, *p =* 0.86
Group 6	No mutations	OR = 1.04, *p =* 0.8	OR = 1.11, *p =* 0.56	OR = 0.69, *p =* 0.07	**OR = 0.24, *p =* 0.003**

The odds ratios (ORs) and *p* values are derived from unconditional logistic regression (Group N versus all other groups except miscellaneous) and are shown for all variables with an association. Clinico-pathological variables with no association (*p >* 0.05) with any group are omitted. Significant associations are shown in bold. WT, wild-type; WD, well differentiated; MD, moderately differentiated; PD, poorly differentiated; F, female; M, male. Group 7 was not tested.

Most CIN^+^ CRCs fell into groups 2 and 3. Although CIN is classically associated with distal location, male gender and higher stage, our two main sets of CIN^+^ cancers (groups 2 and 3) showed distinct differences in their associations (Table 3). Group 2 cancers showed a strong tendency to be distally located and to occur in men, but had no association with stage. Group 3 cancers, on the other hand, were not associated with gender or location, but tended to be stage III tumours. Group 6 cancers were predominantly found in men, for reasons that are unclear. The other three groups showed no association with the clinico-pathological variables, although we note that some groups, such as group 5 (*NRAS*-mutants), were small.

### Association between cancer groups and disease-free survival

**Figure 3:**
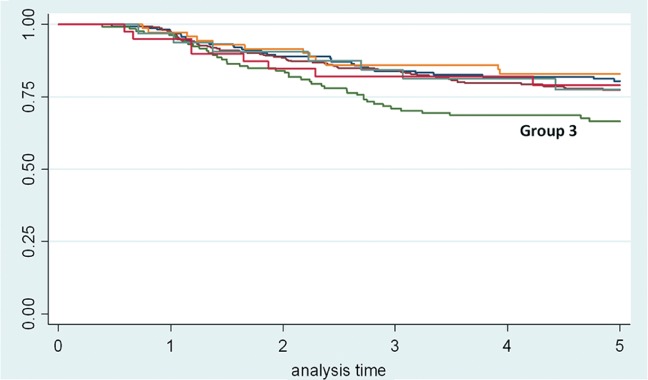
Kaplan–Meier survival curves for disease-free survival (years) of the proposed colorectal cancer groups. Group 3 is KRAS- and/or PIK3CA-mutant; MSI^–^; CIN^+^; TP53 wild-type; BRAF wild-type. A sixth group with no detected mutations is also shown. The seventh residual 'miscellaneous' group was not analysed. Note the only inferior survival is of group 3.

We tested whether our molecular groups of CRC were independent predictors of 5 year disease-free survival in the VICTOR study. All analyses were conditioned on the full set of clinico-pathological variables. We initially analysed group as a categorical variable in the regression model, and we subsequently tested each group against all other groups. Very similar results were obtained in each case. We found that group 3 (*KRAS*- and/or *PIK3CA*-mutant; MSI^–^; CIN^+^; *TP53*-wild-type) patients had poor survival, whereas all the other cancer groups showed no significant differences in survival (hazard ratio = 1.59, 95% CI 1.13–2.24, *p =* 0.008; Cox proportional hazards, group 3 versus all other groups; Figure 3). The only other independent predictor of survival was stage (hazard ratio = 1.99, *p =* 9.0 × 10^–6^, stage III versus stage II). Although group 1 was not an independent predictor of survival, we wondered whether this negative result was caused by some *BRAF*-mutant, MSI^–^ cancers having poor survival 24. We therefore included MSI status in the survival model, since this variable has consistently been associated with better outcome in several studies. Group 3 (hazard ratio = 1.48, 95% CI 1.05–2.09, *p =* 0.027) and stage (hazard ratio = 1.98, *p =* 1.2 × 10^–5^) remained independent predictors of poor prognosis, although MSI was a borderline significant indicator of good prognosis (hazard ratio = 0.59, *p =* 0.054).

## Discussion

Colorectal carcinogenesis follows a multistep model in which sequential molecular alterations occur throughout tumour progression 25. Here, we have analysed most of the common somatic mutations in a large CRC patient set with high-quality clinical trial data. We have undertaken multivariate regression, cluster analysis and Bayesian network analysis that have consistently identified the primary positive and negative associations between molecular changes, thus showing other associations to be indirect. The identification of loops in the pairwise association relationships (Figure 1A) was of particular interest. One such loop provided the basis for explaining the previously-postulated negative association between *TP53* and *KRAS* mutation, which we have now confirmed 7–9,17,20–25,28. One reason why the *KRAS–TP53* association had not found wide acceptance is that it has seemed contrary to the well-established indirect association linking *TP53*–CIN–MSI–*BRAF–KRAS*. Our data show that there is indeed an indirect positive association between *TP53* and *KRAS* mutations that follows this route, but that this is outweighed by a direct negative association. One potential explanation for the negative association lies in the transcription of genes such as *CDKN1A* (p21) by mutant K-ras through p53-dependent and -independent mechanisms. Effects on cell cycle arrest, senescence and apoptosis might sometimes be suboptimal for tumour growth if both genes are mutated.

We confirmed that a small group of double-positive (MSI^+^CIN^+^) CRCs exists, as does a larger group of double-negative (MSI^–^CIN^–^) cancers. The latter were generally similar to MSI^–^CIN^+^ lesions, but had a much lower frequency of *TP53* mutations and lower stage, although relatively high frequencies of *KRAS* and *PIK3CA* mutations. Whilst these findings support *TP53* mutations in some way causing or being permissive for CIN, we found no quantitative differences in ploidy between *TP53*-mutant and *TP53*-wild-type CIN^+^ tumours (data not shown), suggesting that an alternative (epi)mutation to *TP53* inactivation may exist in the latter case.

Uniquely among the molecular changes, *FBXW7/CDC4* mutations occurred randomly in CRC, irrespective of clinico-pathological or other molecular features. Very few other studies have addressed the issue of *FBXW7*'s position in the pathways of colorectal tumourigenesis, apart from functional studies linking its mutation to CIN 27, but we find no evidence of an association with CIN here. One possible explanation for the absence of associations with *FBXW7* is that it mutates early in tumorigenesis, consistent with studies that have found *FBXW7* mutations in colorectal adenomas 30, 31. Fitting (epi)mutations into genetic pathways is likely to become increasingly difficult as next-generation sequencing discovers more low-frequency mutations, such as *FBXW7*, that drive tumourigenesis but do not obviously fit into any specific molecular pathway.

We have proposed a molecular classification of CRC into seven groups (Figure 2), based solely on primary positive and negative associations between molecular changes (MSI, CIN, *TP53* and the type of Ras pathway mutation). This classification essentially retains the MSI^+^ set of cancers as a separate group, but with the addition of *BRAF*-mutant MSI^–^ cancers. We remain open-minded as to whether the latter cancers are distinct from their MSI^+^ counterparts, as some have suggested. Our classification split the CIN^+^ cancers into two groups and established a further *KRAS*- and/or *PIK3CA*-mutant, CIN^–^, *TP53*-wild-type group. Despite our classification being entirely independent of clinico-pathological variables, it showed interesting associations with clinical variables. Our data suggested that the often-reported associations between CIN and gender, tumour location and stage actually comprised two separate associations: (a) between gender, distal location and group 2 tumours (CIN^+^ and/or *TP53*- mutant with wild-type *KRAS* and *PIK3CA*); and (b) between stage III and group 3 tumours (*KRAS*- and/or *PIK3CA*-mutant, CIN^+^, *TP53*-wild-type). These findings cannot readily be explained by differences in sample size and hence statistical power (Table). Group 3, moreover, was an independent predictor of survival, having worse prognosis than any of the other groups. Interestingly, *TP53* mutation is not an established prognostic marker in CRC, despite its strong associations with stage and MSI, and our finding that CIN^+^ *TP53*-wild-type cancers have the poorest prognosis is consistent with this. Furthermore, Hutchins *et al* 28 had previously found poorer disease-free survival in *KRAS*-mutant stage II/III CRCs from the QUASAR trial, although they only performed a univariate analysis. There was also some evidence in our data that MSI might additionally be an independent survival predictor.

Overall, our results show that sufficiently large and homogeneous sample sets and methods based on multivariate and cluster analysis can allow the genetic pathways of cancer to be teased apart for a relatively well-characterized tumour such as CRC. However, this is a complex task that may become even more difficult as next-generation sequencing discovers more low-frequency mutations, such as *FBXW7*, that drive tumorigenesis but do not obviously fit into any specific molecular pathway. However, such studies may provide additional insights through different analyses, one example being the identification of hypermutant, yet MSI^–^, CRCs in a recent large, landmark exome sequencing study 29. Our proposed groups of CRC require replication and refinement, but our data already suggest that a finer-scale molecular classification of CRCs is both possible and desirable, and we expect that a more complex, validated classification of CRCs will emerge gradually in the next few years.

## SUPPLEMENTARY MATERIAL ON THE INTERNET

The following supplementary material mat be found in the online version of this article:

**Supplementary methods**

Figure S1. Spectrum of mutations found in *KRAS*, *NRAS*, *BRAF*, *PIK3CA* and *FBXW7* (A) and TP53.

Table S1. Summary of associations between molecular and clinico-pathological data.

Table S2. Pairwise associations between molecular changes.

Table S3. Logistic regression analysis: summary of significant results for molecular changes.

Table S4. Meta-analysis of studies reporting both *KRAS* and *TP53* mutations.

## References

[b1] Lengauer C, Kinzler KW, Vogelstein B (1997). Genetic instability in colorectal cancers. Nature.

[b2] Ionov Y, Peinado MA, Malkhosyan S (1993). Ubiquitous somatic mutations in simple repeated sequences reveal a new mechanism for colonic carcinogenesis. Nature.

[b3] Walther A, Johnstone E, Swanton C (2009). Genetic prognostic and predictive markers in colorectal cancer. Nat Rev Cancer.

[b4] Sinicrope FA, Rego RL, Halling KC (2006). Prognostic impact of microsatellite instability and DNA ploidy in human colon carcinoma patients. Gastroenterology.

[b5] Toyota M, Ahuja N, Ohe-Toyota M (1999). CpG island methylator phenotype in colorectal cancer. Proc Natl Acad Sci USA.

[b6] Weisenberger DJ, Siegmund KD, Campan M (2006). CpG island methylator phenotype underlies sporadic microsatellite instability and is tightly associated with *BRAF* mutation in colorectal cancer. Nat Genet.

[b7] Peinado M, Fernandezrenart M, Capella G (1993). Mutations in the *p53* suppressor gene do not correlate with *C-k-ras* oncogene mutations in colorectal cancer. Int J Oncol.

[b8] Smith G, Carey FA, Beattie J (2002). Mutations in *APC**Kirsten-ras*, and *p53* – alternative genetic pathways to colorectal cancer. Proc Natl Acad Sci USA.

[b9] Iacopetta B (2003). *TP53* mutation in colorectal cancer. Hum Mut.

[b10] Jass JR (2007). Classification of colorectal cancer based on correlation of clinical, morphological and molecular features. Histopathology.

[b11] Ostwald C, Linnebacher M, Weirich V, Prall F (2009). Chromosomally and microsatellite stable colorectal carcinomas without the CpG island methylator phenotype in a molecular classification. Int J Oncol.

[b12] Rowan A, Halford S, Gaasenbeek M (2005). Refining molecular analysis in the pathways of colorectal carcinogenesis. Clin Gastro Hepatol.

[b13] Midgley RS, McConkey CC, Johnstone EC (2010). Phase III randomized trial assessing rofecoxib in the adjuvant setting of colorectal cancer: final results of the VICTOR trial. J Clin Oncol.

[b14] Samuels Y, Wang Z, Bardelli A (2004). High frequency of mutations of the *PIK3CA* gene in human cancers. Science.

[b15] Segditsas S, Sieber OM, Rowan A (2008). Promoter hypermethylation leads to decreased *APC* mRNA expression in familial polyposis and sporadic colorectal tumours, but does not substitute for truncating mutations. Exp Mol Pathol.

[b16] Vaughn CP, Zobell SD, Furtado LV (2011). Frequency of *KRAS**BRAF*, and *NRAS* mutations in colorectal cancer. Genes Chrom Cancer.

[b17] Wood LD, Parsons DW, Jones S (2007). The genomic landscapes of human breast and colorectal cancers. Science.

[b18] Esteller M, Hamilton SR, Burger PC (1999). Inactivation of the DNA repair gene O6-methylguanine-DNA methyltransferase by promoter hypermethylation is a common event in primary human neoplasia. Cancer Res.

[b19] Jones AM, Douglas EJ, Halford SE (2005). Array-CGH analysis of microsatellite-stable, near-diploid bowel cancers and comparison with other types of colorectal carcinoma. Oncogene.

[b20] Calistri D, Rengucci C, Seymour I (2005). Mutation analysis of *p53**K-ras*, and *BRAF* genes in colorectal cancer progression. J Cell Physiol.

[b21] De Bruijn MT, Raats DAE, Tol J Combined *KRAS* and *TP53* mutation status is not predictive in CAPOX-treated metastatic colorectal cancer. Anticancer Res.

[b22] Giaretti W, Venesio T, Sciutto A (2003). Near-diploid and near-triploid human sporadic colorectal adenocarcinomas differ for *KRAS2* and *TP53* mutational status. Genes Chrom Cancer.

[b23] Reid JF, Gariboldi M, Sokolova V (2009). Integrative approach for prioritizing cancer genes in sporadic colon cancer. Genes Chrom Cancer.

[b24] Samowitz WS, Slattery ML, Sweeney C (2007). *APC* mutations and other genetic and epigenetic changes in colon cancer. Mol Can Res.

[b25] Suehiro Y, Wong CW, Chirieac LR (2008). Epigenetic–genetic interactions in the APC/WNT, RAS/RAF, and P53 pathways in colorectal carcinoma. Clin Can Res.

[b26] Samowitz WS, Sweeney C, Herrick J (2005). Poor survival associated with the *BRAF V600E* mutation in microsatellite-stable colon cancers. Cancer Res.

[b27] Vogelstein B, Fearon ER, Hamilton SR (1988). Genetic alterations during colorectal tumor development. N Eng J Med.

[b28] Bass AJ, Lawrence MS, Brace LE (2011). Genomic sequencing of colorectal adenocarcinomas identifies a recurrent *VTI1A–TCF7L2* fusion. Nat Genet.

[b29] Rajagopalan H, Jallepalli PV, Rago C (2004). Inactivation of hCDC4 can cause chromosomal instability. Nature.

[b30] Hutchins G, Southward K, Handley K (2011). Value of mismatch repair, *KRAS*, and *BRAF* mutations in predicting recurrence and benefits from chemotherapy in colorectal cancer. J Clin Oncol.

[b31] Network CancerGenomeAtlas (2012). Comprehensive molecular characterization of human colon and rectal cancer. Nature.

