# Supplementary material for: Use of multivariate analysis to suggest a new molecular classification of colorectal cancer
Source: J Pathol. 2013 Jan 25;229(3):441–8. doi: 10.1002/path.4139 (PMC3588155; doi:10.1002/path.4139)
Supplement: Figure S1. — Spectrum of mutations found in KRAS, NRAS, BRAF, PIK3CA and FBXW7 (A) and TP53. [file path0229-0441-FigureS1.pdf]

Supplementary Figure 1. Spectrum of mutations found in *KRAS*, *NRAS*, *BRAF*, *PIK3CA* and *FBXW7* (A) and TP53 (B). Non-missense includes nonsense, insertions, deletions and splice site mutations. Aminoacids with hotspot mutations in *TP53* are shown. WT: wild type. [1]One tumour had two concomitant mutations in *PIK3CA*.

A.

KRAS	**N**	***NRAS***	**N**	***BRAF***	**N**	***PIK3CA***	**N**	***FBXW7***	**N**
G12A	16	G12C	2	D594G	2	E542K	15	G423V	2
G12C	28	G12D	10	D594N	2	E542Q	2	I434Del*3	1
G12D	109	G12V	1	V590A	1	I543N	1	R441Q	1
G12P	2	G13D	1	V597T	1	E545D	1	W446X	2
G12R	3	G13V	1	V600E	84	E545G	4	R465C	9
G12S	15	Q61H	1	S602Y	1	E545K	37	R465H	10
G12V	65	Q61K	9	WT	812	E545R	1	R473G	1
G13D	56	Q61L	1			Q546H	1	R473Del*1	1
G13R	1	Q61R	6			Q546K	8	IVS8 +1	2
G13S	4	WT	837			Q546R	4[1]	R479S	1
Q61H	1					M1043V	1	R479P	1
Q61R	2					D1045N	1[1]	D480H	1
A146S	1					H1047R	19	R505C	1
A146T	1					H1047Y	3	R505H	1
WT	594					H1048T	1	H535Del*1	1
						G1049A	2	WT	715
						G1049D	1		
						G1049R	2		
						G1049S	1		
						WT	792		

B.

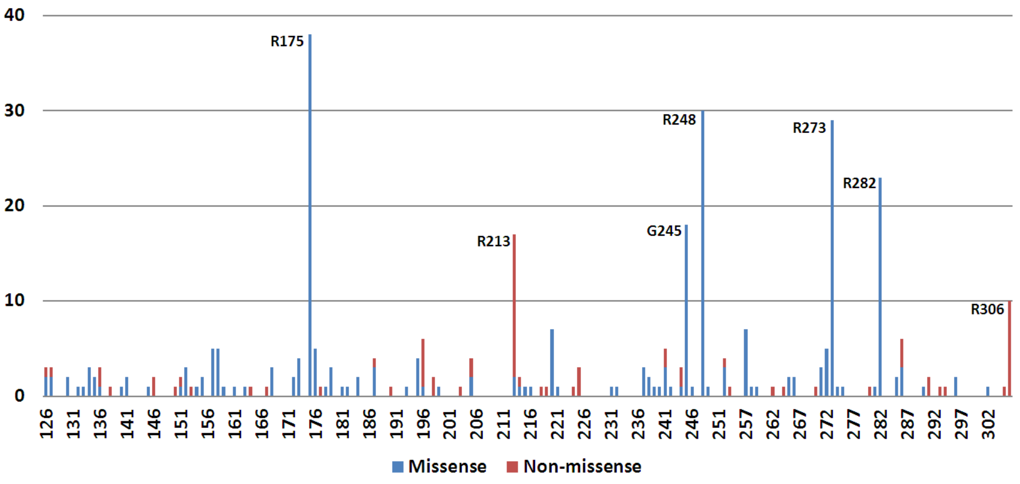

For Peer Revi
